# Nutritional literacy profiles and their association with dietary motivation in adolescents with chronic kidney disease

**DOI:** 10.3389/fnut.2026.1965268

**Published:** 2026-10-15

**Authors:** Shuo Bai, Jiwei Qi, Wuyue Xu, Chen Rong, Lili Jia

**Affiliations:** Department of Pediatrics, Jinling Hospital, Affiliated Hospital of Medical School, Nanjing University, Nanjing, China

**Keywords:** adolescents, chronic kidney disease, dietary motivation, influencing factors, latent profile analysis, nutritional literacy

## Abstract

**Objective:**

To explore potential categories of nutritional literacy among adolescents with chronic kidney disease and differences in dietary motivations across these categories.

**Methods:**

This cross-sectional study included 433 adolescents with chronic kidney disease treated in the pediatric department of a tertiary hospital between March and September 2025. Latent profile analysis identified nutrition literacy profiles, and dietary motivation scores were compared across profiles. Multinomial logistic regression examined associations between participant characteristics and profile membership, using the moderate-literacy profile as the reference. Adjusted odds ratios and 95% confidence intervals were reported.

**Results:**

Nutritional literacy among adolescents with chronic kidney disease can be divided into three latent categories: Category 1 is the “high nutrition literacy” comprising 159 participants (36.8%); Category 2 is the “moderate nutrition literacy” comprising 95 participants (21.9%); Category 3 is the “low nutrition literacy” comprising 179 participants (41.3%). Comparisons of total dietary motivation scores among adolescents with chronic kidney disease in different nutritional literacy categories revealed statistically significant differences (*p* < 0.05). Logistic regression analysis results indicated that gender, being an only child, monthly income, and method of medical payment were independent factors influencing the latent categories of nutritional literacy among adolescents with chronic kidney disease (*p* < 0.05).

**Conclusion:**

There is heterogeneity in nutritional literacy among adolescents with chronic kidney disease, and patients in different nutritional literacy categories exhibit varying levels of dietary motivation. Healthcare professionals should develop personalized intervention strategies based on patients’ nutritional literacy categories to improve their nutritional literacy and beliefs regarding healthy eating.

## Background

Chronic kidney disease (CKD) is a chronic condition that seriously affects the physical and mental health of children and adolescents (1). With advances in diagnostic and therapeutic technologies, the survival rates of adolescents with CKD have continued to improve; however, the long-term management of the disease remains a significant challenge (2). Dietary management is a key component of comprehensive CKD treatment; proper control of the intake of nutrients such as protein, sodium, potassium, and phosphorus helps maintain patients’ nutritional status and slow disease progression (3). Adolescent patients are at a critical stage of physical and psychological development. The conflict between their dietary needs and disease-related restrictions, combined with factors such as insufficient self-management skills and lack of family support, negatively affects their adherence to dietary management (4). Nutrition literacy is an important component of health literacy; it refers to an individual’s ability to access, understand, evaluate, and apply nutrition information to guide healthy dietary decisions (5). A high level of nutritional literacy helps promote improvements in patients’ dietary behaviors and disease management. Current research on nutritional literacy among patients with chronic kidney disease (CKD) has primarily focused on adults, with most studies examining overall levels and associated factors; however, there is a scarcity of research on nutritional literacy among adolescent CKD patients. Dietary motivation, as a key psychological factor driving individuals to maintain healthy dietary behaviors, influences the translation of nutritional knowledge into actual dietary behaviors; yet, the relationship between the two has not been thoroughly investigated (6). Latent profile analysis (LPA) can identify different subgroups of individuals based on their characteristics, reveal heterogeneity within these groups, and provide new research directions for precision nursing interventions. Therefore, this study employs latent profile analysis to explore the latent category characteristics of nutritional literacy among adolescent CKD patients and to analyze the relationship between different nutritional literacy categories and dietary motivation, with the aim of providing a theoretical basis for the development of individualized nutritional education and dietary management strategies in clinical practice.

## Methods

### Objectives of the study

From March 2025 to September 2025, 433 pediatric patients with kidney disease were selected as study subjects from the Department of Pediatrics at the General Hospital of the Eastern Theater Command. Inclusion criteria: (1) met the diagnostic criteria for chronic kidney disease (7); (2) aged 10–18 years; (3) both the patient and the guardian provided informed consent to participate in this study. Exclusion criteria: (1) Concurrent severe cardiopulmonary or other organ failure; (2) Concurrent malignant tumors; (3) Severe illness making participation in the study difficult; (4) Hearing or speech communication impairments. According to Kendall’s rule, the sample size should be 10–20 times the number of independent variables. This study initially identified 18 independent variables; factoring in a 20% rate of invalid questionnaires, the estimated required sample size was ≥216 cases. Potential cross-sectional analysis suggests a sample size of ≥300 cases. Ultimately, 433 subjects meeting the inclusion criteria were enrolled. The study was approved by the Ethics Committee of the General Hospital of the Eastern Theater Command on 6 March 2025 (approval No. 2025DZGJJ-026).

### Investigation content relevant to this study

Questionnaire on General Demographic Information for Adolescents with Kidney Disease: gender, age, whether an only child, place of residence, monthly household income, and method of medical payment.

Questionnaire on Medical History for Adolescents with Kidney Disease: type of disease, duration since diagnosis, number of hospitalizations, treatment methods, and symptoms.

Sociodemographic and clinical variables. Monthly household income was reported by the parent or guardian as the total income received by all household members in a typical month, in Chinese yuan (CNY). It was categorized as <3,000, 3,000–5,000, or >5,000 CNY per month. Information on treatment and symptoms was obtained from the patients’ medical records. Treatment was classified into four mutually exclusive categories according to the treatment documented at the time of the survey: Hormonese, glucocorticoids plus immunosuppressants, glucocorticoids plus immunosuppressants plus biologic agents, and other treatments. Hematuria, proteinuria, and the absence of recorded symptoms were classified as clinical manifestations and were not treated as treatment categories. The primary method of paying medical expenses was classified as medical insurance, self-payment, or other payment methods.

Dietary Motivation Questionnaire: Developed by Pelletier et al. and adapted into Chinese by Di Lihua, this questionnaire is used to assess the level of motivation for healthy eating behaviors (8). The questionnaire comprises five dimensions—intrinsic motivation, integrative regulation, cognitive regulation, introjective regulation, and extrinsic regulation—with a total of 20 items. Each item uses a 7-point Likert scale, ranging from “Strongly Disagree” to “Strongly Agree” and scored from 1 to 7. The total score ranges from 20 to 140; a higher score indicates stronger motivation for healthy eating. The Cronbach’s α coefficient for the total questionnaire is 0.851.

Chinese Version of the Nutrition Literacy Assessment Tool: This scale was localized and validated by Chinese scholars, including Chen Yuanyuan, et al. (9). It comprises six dimensions: Nutrition and Health, Energy Sources in Food, Household Food Measurement, Food Label Calculations, Food Categories, and Consumption Skills. The scale consists of a total of 38 items; correct answers are scored as 1 point, and incorrect answers as 0 points, resulting in a total score ranging from 0 to 38 points. The Cronbach’s α coefficient for this scale is 0.860.

### Statistical methods

Data analysis was performed using SPSS 26.0 software, and latent profile analysis was conducted using Mplus 8.3 software. Quantitative data that followed a normal distribution were expressed as mean ± standard deviation (x̄ ± s), and comparisons between groups were performed using the independent samples t-test or analysis of variance (ANOVA); data that did not follow a normal distribution were expressed as median (interquartile range), and comparisons between groups were performed using nonparametric tests; Categorical data are presented as frequencies and percentages (%), and comparisons between groups were performed using the χ^2^ test. A *p*-value < 0.05 was considered statistically significant. Latent profile analysis was used to identify latent categories of nutritional literacy among adolescents with CKD. Using scores on each dimension of nutritional literacy as manifest variables, models with 1 to 4 latent categories were established and compared for fit. Model fit metrics included: Akaike Information Criterion (AIC), Bayesian Information Criterion (BIC), and sample-corrected Bayesian Information Criterion (aBIC); lower values indicate better model fit; entropy was used to evaluate classification accuracy, with values closer to 1 indicating higher classification quality. The optimal latent category model was determined by comprehensively considering model fit metrics, category proportions, statistical significance, and clinical interpretability.

## Results

### General information on adolescents with kidney disease

This study included a total of 433 patients, of whom 248 were male (57.1%) and 186 were female (42.9%); 314 patients (72.4%) were aged 10–15, and 120 (27.6%) were aged 16–18; patients’ nutrition literacy scores ranged from 4 to 36 points, with a mean score of (20.36 ± 4.28) points.

### An analysis of the potential profile of nutritional literacy among adolescents with kidney disease

Based on the scores across the five dimensions of the nutritional literacy assessment questionnaire for adolescents with kidney disease, we constructed models with 1 to 4 latent categories and conducted goodness-of-fit tests. The results showed that as the number of latent categories increased, AIC, BIC, and aBIC generally decreased. Among these, Model 4 had the lowest AIC and aBIC; however, its LMRT did not reach the significance threshold (*p* > 0.05), indicating that adding a fourth latent category did not significantly improve the model’s explanatory power. Model 3 had a higher entropy value than Model 2; furthermore, both the LMRT and BLRT tests for Model 3 were statistically significant (both *p* < 0.001). After comprehensive consideration, three latent categories of nutritional literacy among adolescents with kidney disease were selected for analysis, as shown in Table 1.

**Table 1:** Model fit metrics for different categories of nutritional literacy.

Model	AIC	BIC	aBIC	Entropy	LMRT P	BLRT P	Category probability
1.	13853.5	13894.2	13862.4	—	—	—	1.000
2.	12830.9	12896.1	12845.3	0.882	<0.001	<0.001	0.647/0.353
3.	12480.8	12570.4	12500.6	0.907	<0.001	<0.001	0.413/0.219/0.368
4.	12360.8	12474.8	12385.9	0.846	0.333	<0.001	0.373/0.171/0.266/0.190

AIC, Akaike information criterion; BIC, Bayesian information criterion; aBIC, sample-size adjusted Bayesian information criterion; LMRT, Lo–Mendell–Rubin likelihood-ratio test; BLRT, bootstrap likelihood-ratio test.

### Characteristics and nomenclature of potential categories of nutritional literacy among adolescents with kidney disease

Based on the results of the latent profile analysis, it was found that adolescents with kidney disease can be classified into three categories. By plotting line graphs of the scores for each dimension of the nutritional literacy scale (Figure 1), the characteristics of nutritional literacy among adolescents with kidney disease in these three latent categories can be analyzed more clearly. The three latent categories were named as follows: Category 1 is the “high nutrition literacy” comprising 159 participants (36.8%); Category 2 is the “moderate nutrition literacy” comprising 95 participants (21.9%); Category 3 is the “low nutrition literacy” comprising 179 participants (41.3%).

**Figure 1:**
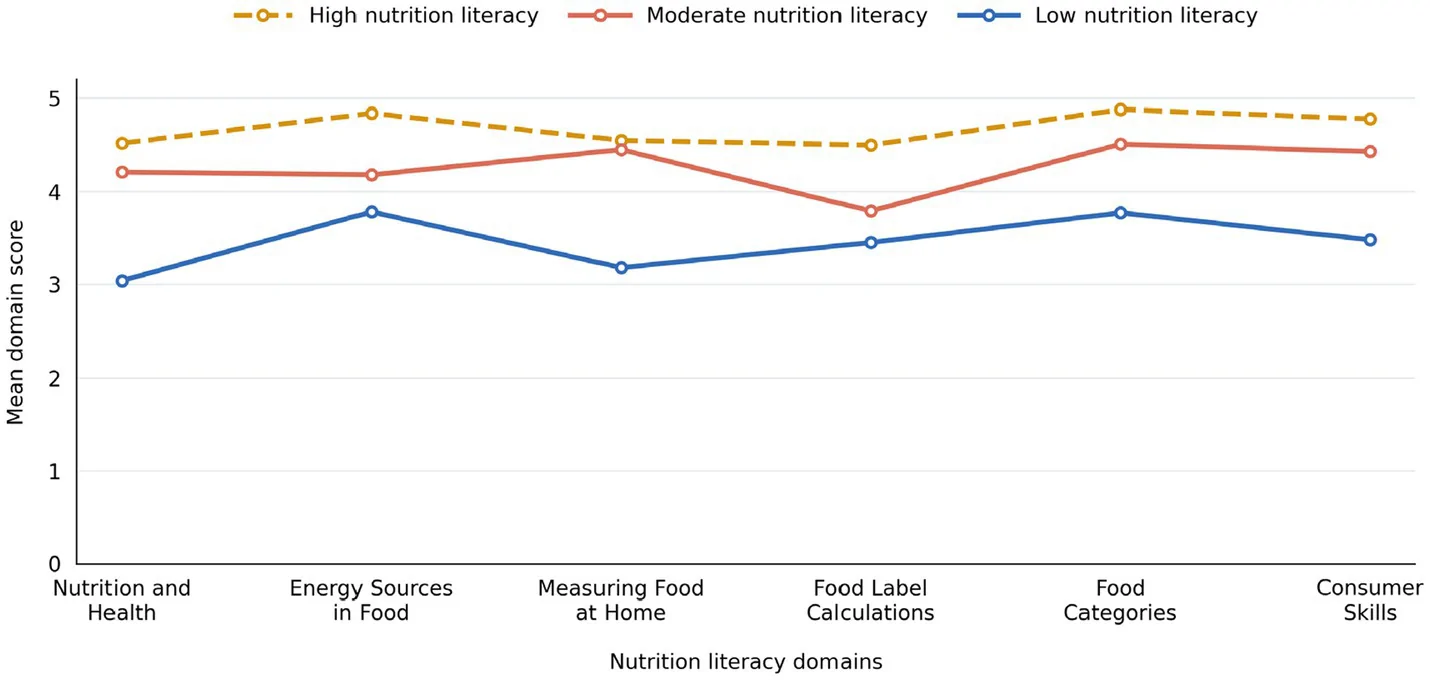
Nutrition literacy domain scores across three latent profiles. The horizontal axis shows six nutrition literacy domains; the vertical axis shows mean domain scores. The three lines represent the high, moderate, and low profiles. Plotted values were estimated from the original image and must be replaced with exact group means before submission.

A Comparison of Dietary Motivation Scores Among Adolescents with Kidney Disease Across Different Potential Categories of Nutritional Literacy.

There were statistically significant differences among the three groups in terms of dietary behavior, physical activity, cognition and attitudes, psychological coping,

social support, and total compliance scores (*p* < 0.05); see Table 2.

**Table 2:** Comparison of dietary motivation scores by nutritional literacy level (x ± s).

Dimension	High nutrition literacy *n* = 159	moderate nutrition literacy *n* = 95	low nutrition literacy n = 179	F	*p*
Eating behaviors	33.98 ± 7.89	27.19 ± 8.63	25.61 ± 6.57	55.222	<0.001
Physical activity	21.45 ± 4.97	21.07 ± 5.05	17.87 ± 5.45	22.516	<0.001
Perceptions and attitudes	24.19 ± 4.97	22.89 ± 5.74	18.56 ± 4.43	56.789	<0.001
Mental adjustment	18.71 ± 4.66	17.85 ± 5.22	12.79 ± 4.41	72.927	<0.001
Social support	22.21 ± 4.81	20.94 ± 5.37	17.25 ± 4.16	48.451	<0.001
Total score	126.25 ± 23.12	121.47 ± 24.97	119.43 ± 17.29	4.387	0.013

### Participant characteristics by nutrition literacy profile

Participant characteristics across the three nutrition literacy profiles are summarized in Table 3.

**Table 3:** Participant characteristics by nutrition literacy profile (*n* = 433).

Variables	High nutrition literacy *n* = 159	Moderate nutrition literacy *n* = 95	Low nutrition literacy *n* = 179
Sex			
Female	61 (38.4)	59 (62.1)	48 (26.8)
Male	98 (61.6)	36 (37.9)	131 (73.2)
A single birth			
Yes	58 (36.5)	20 (21.1)	63 (35.2)
No	101 (63.5)	75 (78.9)	116 (64.8)
Length of diagnosis			
≤12 months	45 (28.3)	37 (38.9)	39 (21.8)
13-24 months	21 (13.2)	16 (16.8)	22 (12.3)
>24 months	93 (58.5)	42 (44.2)	118 (65.9)
Frequency of hospitalization			
<5	122 (76.7)	73 (76.8)	135 (75.4)
≥5	37 (23.3)	22 (23.2)	44 (24.6)
Type of treatment			
Hormones	75 (47.2)	38 (40.0)	73 (40.8)
Hormones + immunosuppressants	29 (18.2)	25 (26.3)	42 (23.5)
Hormones + immunosuppressants + biologicals	9 (5.7)	10 (10.5)	11 (6.1)
Other	46 (28.9)	22 (23.2)	53 (29.6)
Symptomatic			
Hematuria	88 (55.3)	51 (53.7)	93 (52.0)
Proteinuria	15 (9.4)	10 (10.5)	18 (10.1)
Asymptomatic	56 (35.2)	34 (35.8)	68 (38.0)
Place of residence			
City	101 (63.5)	63 (66.3)	95 (53.1)
Rural	58 (36.5)	32 (33.7)	84 (46.9)
Monthly household income(CNY)			
<3,000	50 (31.4)	26 (27.4)	78 (43.6)
3,000–5,000	44 (27.7)	29 (30.5)	46 (25.7)
>5,000	65 (40.9)	40 (42.1)	55 (30.7)
Medical payment methods			
Medical insurance	76 (47.8)	36 (37.9)	79 (44.1)
Self-financed	67 (42.1)	37 (38.9)	93 (52.0)
Other	16 (10.1)	22 (23.2)	7 (3.9)

Values are *n* (%). Percentages were calculated within each profile using the sample sizes shown in the column headings and rounded to one decimal place. CNY, Chinese yuan.

#### Factors associated with nutrition literacy profile membership

With the moderate-literacy profile as the reference outcome category, multinomial logistic regression identified several characteristics associated with nutrition literacy profile membership. In the high-versus-moderate comparison, males had lower adjusted odds of high-literacy profile membership than females (aOR = 0.570, 95% CI: 0.355–0.915; *p* = 0.020). Participants with a monthly household income of 3,000–5,000 CNY had higher adjusted odds of high-literacy profile membership than those with an income below 3,000 CNY (aOR = 1.759, 95% CI: 1.039–2.978; *p* = 0.036). Participants using other payment methods also had higher adjusted odds of high-literacy profile membership than those using medical insurance (aOR = 2.928, 95% CI: 1.121–7.646; *p* = 0.028). No statistically significant associations were observed for the remaining predictor levels in this comparison (Table 4).

**Table 4:** Adjusted associations between participant characteristics and high-literacy versus moderate-literacy profile membership.

Predictor contrast	High vs. moderate literacy	
	aOR (95% CI)	*p* value
Sex: male vs. female	0.570 (0.355–0.915)	0.020
Only-child status: no vs. yes	1.041 (0.654–1.655)	0.866
Time since diagnosis: 13–24 vs. ≤ 12 months	0.664 (0.393–1.122)	0.126
Time since diagnosis: >24 vs. ≤ 12 months	0.802 (0.406–1.587)	0.527
Residence: rural vs. urban	0.638 (0.406–1.005)	0.053
Monthly household income: 3,000–5,000 vs. < 3,000 CNY	1.759 (1.039–2.978)	0.036
Monthly household income: >5,000 vs. < 3,000 CNY	1.184 (0.672–2.085)	0.560
Payment method: self-payment vs. medical insurance	2.212 (0.849–5.767)	0.104
Payment method: other vs. medical insurance	2.928 (1.121–7.646)	0.028

aOR, adjusted odds ratio; CI, confidence interval; CNY, Chinese yuan. The moderate-literacy profile is the reference outcome category. Predictor reference categories are specified after “vs” in the first column. Estimates are from the multinomial logistic regression model described in the manuscript.

In the low-versus-moderate comparison, males had higher adjusted odds of low-literacy profile membership than females (aOR = 2.713, 95% CI: 1.566–4.702; *p* < 0.001). Participants who were not an only child had lower adjusted odds of low-literacy profile membership than those who were an only child (aOR = 0.446, 95% CI: 0.239–0.832; *p* = 0.011). Compared with medical insurance, self-payment (aOR = 0.330, 95% CI: 0.149–0.731; *p* = 0.006) and other payment methods (aOR = 0.353, 95% CI: 0.158–0.792; *p* = 0.012) were associated with lower adjusted odds of low-literacy profile membership. No statistically significant associations were observed for the remaining predictor levels in this comparison (Table 5).

**Table 5:** Adjusted associations between participant characteristics and low-literacy versus moderate-literacy profile membership.

Predictor contrast	Low vs. moderate literacy	
	aOR (95% CI)	*p* value
Sex: male vs. female	2.713 (1.566–4.702)	<0.001
Only-child status: no vs. yes	0.446 (0.239–0.832)	0.011
Time since diagnosis: 13–24 vs. ≤ 12 months	1.761 (0.966–3.213)	0.065
Time since diagnosis: >24 vs. ≤ 12 months	1.701 (0.774–3.736)	0.186
Residence: rural vs. urban	1.179 (0.671–2.070)	0.567
Monthly household income: 3,000–5,000 vs. < 3,000 CNY	0.703 (0.358–1.377)	0.304
Monthly household income: >5,000 vs. < 3,000 CNY	1.001 (0.519–1.931)	0.997
Payment method: self-payment vs. medical insurance	0.330 (0.149–0.731)	0.006
Payment method: other vs. medical insurance	0.353 (0.158–0.792)	0.012

aOR, adjusted odds ratio; CI, confidence interval; CNY, Chinese yuan. The moderate-literacy profile is the reference outcome category. Predictor reference categories are specified after “vs” in the first column. Estimates are from the multinomial logistic regression model described in the manuscript.

## Discussion

Nutritional literacy is an individual’s ability to access, understand, evaluate, and apply nutritional information to support healthy dietary decisions; it is an important manifestation of health literacy in the field of nutrition management (10). For adolescents with chronic kidney disease, dietary management is an integral part of the entire treatment process. Appropriately controlling the intake of nutrients such as protein, sodium, potassium, and phosphorus not only helps maintain their normal nutritional status but also reduces the risk of disease progression (11). The results of this study show that the overall nutritional literacy scores among adolescents with CKD are generally low, suggesting that this population still has significant deficiencies in nutritional knowledge, information interpretation, and dietary decision-making abilities. Adolescence is a critical period for physiological development and the formation of behavioral patterns, during which dietary choices are influenced by multiple factors, including family eating habits and self-control (12). Adolescents with CKD face long-term limitations due to their condition and must strike a balance between their growth and developmental needs and dietary management for their disease; the high level of dietary complexity may increase the difficulty they face in understanding and applying nutritional information (13). Previous nutrition education has largely focused on educating patients about diseases, but has failed to cultivate their ability to access nutritional information, read food labels, and manage their diets in practice. This may result in patients understanding some dietary requirements for certain diseases but struggling to translate that knowledge into daily behavior (14). Using latent profile analysis, this study further found that nutritional literacy among adolescents with CKD does not follow a single continuous distribution but exhibits distinct categorical differences, allowing for classification into high, low, and moderate nutritional literacy groups. The low nutritional literacy group accounted for the highest proportion, suggesting that clinical healthcare providers should avoid a “one-size-fits-all” educational approach when implementing nutritional management and instead adopt a tiered management strategy based on patients’ levels of nutritional literacy. For patients with low nutritional literacy, the focus should be on strengthening their basic nutritional knowledge and dietary skills; for patients with moderate and high nutritional literacy, efforts should be directed toward further enhancing their disease self-management abilities and improving the quality of their nutritional decision-making.

The results of this study show that patients in different potential categories of nutritional literacy exhibit significant differences across dimensions related to dietary motivation, including dietary behavior, physical activity, cognition and attitudes, psychological coping, and social support. This suggests that the level of nutritional literacy serves as an internal driver influencing the formation of patients’ dietary behaviors. Dietary behavior depends not only on whether patients possess diet-related knowledge but is also influenced by factors such as behavioral motivation, health beliefs, and the social environment (15). Patients with higher nutritional literacy possess stronger information-processing abilities and can more easily understand the relationship between dietary restrictions and disease management, thereby developing a more positive attitude toward healthy eating and a greater willingness to adopt healthy dietary behaviors. In contrast, patients with insufficient nutritional literacy are unable to accurately recognize the importance of dietary management and find it difficult to establish disease management goals, resulting in a lack of motivation to adhere to dietary behaviors (16). This study also found that patients in the low nutritional literacy group had lower overall levels of dietary motivation, although scores on certain dimensions indicated a certain degree of inclination toward healthy behaviors. This suggests that simply improving patients’ nutritional knowledge is not sufficient to fully promote changes in their dietary behaviors; further attention must be paid to stimulating patients’ psychological needs and behavioral motivation. Therefore, clinical nutritional interventions should shift from the traditional knowledge-transfer model to a comprehensive “knowledge–attitude–motivation–behavior” intervention model (17). Nursing staff should incorporate theoretical frameworks such as Self-Determination Theory and the Health Belief Model (18) to enhance patients’ autonomy in dietary management and improve long-term adherence through goal-setting, behavioral feedback, and family support.

Multivariate logistic regression analysis in this study revealed that gender, being an only child, monthly income, and method of medical payment are significant factors influencing the potential categories of nutritional literacy among adolescents with CKD. Among these, male patients had a higher risk of falling into the low nutritional literacy group, suggesting that gender may influence adolescents’ attention to disease management and dietary behaviors. Previous studies have shown (19) that female adolescents tend to be more proactive in accessing health information, paying attention to their health, and engaging in healthy behaviors; therefore, special attention should be paid to male patients’ health awareness during nutrition education. The family environment is also a key factor influencing adolescents’ nutritional literacy (20). Families with an only child may have access to more abundant family resources and care, whereas patients from families with multiple children may face the challenge of scattered family care resources. Therefore, healthcare providers should incorporate the family into the nutritional management system and create a favorable dietary management environment for patients by enhancing caregivers’ nutritional knowledge and the family’s support capacity (21). Patients’ nutritional literacy is influenced by economic status and healthcare payment methods (22). Families with better economic conditions have greater access to health resources and can receive more systematic nutritional guidance, whereas families facing heavier financial burdens may focus more on the treatment costs of the disease itself and underinvest in long-term dietary management (23). Therefore, clinical practice should prioritize socioeconomically disadvantaged patients by providing targeted health guidance and integrating medical resources (24) to reduce barriers to nutritional management.

### Limitations

This study has certain limitations. As a single-center cross-sectional survey that primarily collected patient data through self-report questionnaires, it may be subject to some bias. Future research could conduct multicenter, large-sample longitudinal studies that combine objective nutritional assessment indicators with clinical outcome measures to further validate the stability of the potential nutritional literacy categories and their effects on dietary management, thereby providing a basis for developing a precision-based nutritional management model. Dietary motivation was examined through unadjusted comparisons across nutrition literacy profiles rather than through a multivariable model with dietary motivation as the outcome. Consequently, the observed differences may be confounded by demographic, family, socioeconomic, or clinical characteristics, and the independent association between profile membership and dietary motivation cannot be determined from this study. In addition, the cross-sectional design does not establish the temporal order of nutrition literacy and dietary motivation. Future studies should verify the measurement of dietary motivation and use appropriately adjusted models to examine this relationship.

## Conclusion

This study employed latent profile analysis to examine the characteristics of nutritional literacy among adolescents with chronic kidney disease (CKD) and its relationship with dietary motivation. The results showed that there were significant differences in the levels of nutritional literacy among adolescent CKD patients, who could be classified into three latent categories: high nutritional literacy, moderate nutritional literacy, and low nutritional literacy, with the low nutritional literacy group accounting for a relatively high proportion. There were significant differences in dietary motivation among patients in different nutritional literacy categories, suggesting that nutritional literacy is closely related to patients’ healthy dietary behaviors. Further analysis revealed that gender, being an only child, monthly income, and method of medical payment were independent factors influencing patients’ nutritional literacy categories. The findings suggest that clinical nursing staff should pay attention to individual differences in nutritional literacy among adolescent CKD patients, develop stratified and individualized intervention plans based on different levels of nutritional literacy, and combine these with dietary motivation to improve patients’ dietary management abilities and self-management levels.

## Data Availability

The original contributions presented in the study are included in the article/supplementary material, further inquiries can be directed to the corresponding author.

## References

[1] Zhao WM Li XL Shi R Zhu Y Wang ZJ Wang XR . Global, regional and national burden of CKD in children and adolescents from 1990 to 2019. Nephrol Dial Transplant. (2024) 39:1268–78. doi: 10.1093/ndt/gfad269, 38130213

[2] Muniz VM de Paiva Souza Klippel L Freitas KLF dos Santos Barbosa K Libardi MC de Paula Alves Bezerra OM . Clinical complications and anthropometric indicators of chronic kidney disease in a global context: scoping review. Pediatr Nephrol. (2026) 1:7381. doi: 10.1007/s00467-026-07381-9, 42246999 PMC13672459

[3] Luo D Liu L Zhang S Guan Y He J. Nutritional management of chronic kidney disease in children: a best evidence summary. Int Urol Nephrol. (2026) 58:1393–407. doi: 10.1007/s11255-025-04802-w, 41046486

[4] Chen K Didsbury M van Zwieten A Howell M Kim S Tong A . Neurocognitive and educational outcomes in children and adolescents with CKD: a systematic review and meta-analysis. Clin J Am Soc Nephrol. (2018) 13:387–97. doi: 10.2215/CJN.09650917, 29472306 PMC5967677

[5] Akchurin O Molino AR Schneider MF Atkinson MA Warady BA Furth SL. Longitudinal relationship between anemia and statural growth impairment in children and adolescents with nonglomerular CKD: findings from the chronic kidney disease in children (CKiD) study. Am J Kidney Dis. (2023) 81:457–465.e1. doi: 10.1053/j.ajkd.2022.09.019, 36481700 PMC10038884

[6] Zhang A van Zwieten A Hughes A Kim S Lambert K Torrisi LG . Patient and caregiver perspectives on diet and nutrition for children with CKD: a systematic review of qualitative studies. Am J Kidney Dis. (2026) 87:345–363.e1. doi: 10.1053/j.ajkd.2025.09.011, 41238162

[7] Wang L He Y Han C Zhu P Zhou Y Tang R . Global burden of chronic kidney disease and risk factors, 1990–2021: an update from the global burden of disease study 2021. Front Public Health. (2025) 13:1542329. doi: 10.3389/fpubh.2025.1542329, 40843419 PMC12366504

[8] Daly AN O’Sullivan EJ Walton J Kehoe L McNulty BA Flynn A . Determining the food choice motivations of Irish teens and their association with dietary intakes, using the food choice questionnaire. Appetite. (2023) 189:106981. doi: 10.1016/j.appet.2023.106981 37499761

[9] McNamara J Kunicki ZJ Neptune L Parsons K Byrd-Bredbenner C. Development and validation of the young adult nutrition literacy tool. J Nutr Educ Behav. (2022) 54:691–701. doi: 10.1016/j.jneb.2022.01.011, 35469757

[10] Jayasinghe S Byrne NM Hills AP. Cultural influences on dietary choices. Prog Cardiovasc Dis. (2025) 90:22–6. doi: 10.1016/j.pcad.2025.02.003, 39921186

[11] Pal S Panduragan SL Said FM Poddar S. Effect of self-management intervention on improvement of quality of life in chronic kidney disease patients: a scoping review. Open Nurs J. (2024) 18:4442. doi: 10.2174/0118744346323309241009074442

[12] Silva P Araújo R Lopes F Ray S. Nutrition and food literacy: framing the challenges to health communication. Nutrients. (2023) 15:4708. doi: 10.3390/nu15224708, 38004102 PMC10674981

[13] Mak RH Kovesdy CP Hao S Wang AY. Nutrition across the entire lifespan in CKD: from childhood to senescence. Clin J Am Soc Nephrol. (2026) 21:863–71. doi: 10.2215/CJN.0000000786, 40504772 PMC13143487

[14] Kanagaratnam R Zwi S Webster AC Isautier J Lambert K Shepherd HL . Interventions to support decision-making, health literacy and self-management in ethnic-minority adults living with chronic kidney disease: a systematic review. Clin Kidney J. (2023) 16:914–27. doi: 10.1093/ckj/sfac276, 37260990 PMC10228681

[15] Russell CR 3rd Zigan C Wozniak K Soni K Hill Gallant KM Friedman AN. A systematic review and qualitative analysis of existing dietary mobile applications for people with chronic kidney disease. J Ren Nutr. (2022) 32:382–8. doi: 10.1053/j.jrn.2021.06.006, 34334288 PMC8799771

[16] McAuley EA Ross LA Hannan-Jones MT MacLaughlin HL. Diet quality, self-efficacy, and health literacy in adults with chronic kidney disease: a cross-sectional study. J Ren Nutr. (2025) 35:410–8. doi: 10.1053/j.jrn.2024.06.005, 38897366

[17] Zhong Y Patel N Ferris M Rak E. Health literacy, nutrition knowledge, and health care transition readiness in youth with chronic kidney disease or hypertension: a cross-sectional study. J Child Health Care. (2020) 24:246–59. doi: 10.1177/1367493519831493, 30866644

[18] Campbell ZC Dawson JK Kirkendall SM McCaffery KJ Jansen J Campbell KL . Interventions for improving health literacy in people with chronic kidney disease. Cochrane Database Syst Rev. (2022) 2022:CD012026. doi: 10.1002/14651858.CD012026.pub2, 36472416 PMC9724196

[19] Lockwood G Davey L McFarlane C Gray NA Wright HH. Factors influencing meal provision and dietary support behaviour of caregivers of people with chronic kidney disease: a cross-sectional study. Nutrients. (2024) 16:3479. doi: 10.3390/nu16203479, 39458474 PMC11510062

[20] Ruiz LD Zuelch ML Dimitratos SM Scherr RE. Adolescent obesity: diet quality, psychosocial health, and cardiometabolic risk factors. Nutrients. (2020) 12:43. doi: 10.3390/nu12010043, 31877943 PMC7020092

[21] Moin A Schneider MF Matheson MB Warady BA Furth S Akchurin O. Food assistance to financially insecure children and adolescents with CKD: impact on disease progression. J Ren Nutr. (2026) 36:520–3. doi: 10.1053/j.jrn.2025.12.005, 41548623 PMC13180299

[22] Hoteit M Younes H Tayyem R. Editorial on nutrition education, food literacy, and healthy diets in childhood and adolescence. Nutrients. (2024) 16:4108. doi: 10.3390/nu16234108, 39683502 PMC11643460

[23] Hoteit M Qasrawi R Tayyem R. Editorial: food literacy and healthy diets in childhood and adolescence. Front Nutr. (2024) 11:1366912. doi: 10.3389/fnut.2024.1366912, 38332801 PMC10850368

[24] Engel E Gell S Heiss R Karsay K. Social media influencers and adolescents’ health: a scoping review of the research field. Soc Sci Med. (2024) 340:116387. doi: 10.1016/j.socscimed.2023.116387, 38039770

